# Barx1-Mediated Inhibition of Wnt Signaling in the Mouse Thoracic Foregut Controls Tracheo-Esophageal Septation and Epithelial Differentiation

**DOI:** 10.1371/journal.pone.0022493

**Published:** 2011-07-22

**Authors:** Janghee Woo, Isabelle Miletich, Byeong-Moo Kim, Paul T. Sharpe, Ramesh A. Shivdasani

**Affiliations:** 1 Department of Medical Oncology, Dana-Farber Cancer Institute, Boston, Massachusetts, United States of America; 2 Graduate Program in Biological and Biomedical Science, Harvard University, Cambridge, Massachusetts, United States of America; 3 Department of Craniofacial Development, Dental Institute, King's College, London, United Kingdom; 4 Department of Medicine, Brigham and Women's Hospital and Harvard Medical School, Boston, Massachusetts, United States of America; University of Maastricht (UM), The Netherlands

## Abstract

Mesenchymal cells underlying the definitive endoderm in vertebrate animals play a vital role in digestive and respiratory organogenesis. Although several signaling pathways are implicated in foregut patterning and morphogenesis, and despite the clinical importance of congenital tracheal and esophageal malformations in humans, understanding of molecular mechanisms that allow a single tube to separate correctly into the trachea and esophagus is incomplete. The homoebox gene *Barx1* is highly expressed in prospective stomach mesenchyme and required to specify this organ. We observed lower Barx1 expression extending contiguously from the proximal stomach domain, along the dorsal anterior foregut mesenchyme and in mesenchymal cells between the nascent esophagus and trachea. This expression pattern exactly mirrors the decline in Wnt signaling activity in late development of the adjacent dorsal foregut endoderm and medial mainstem bronchi. The hypopharynx in *Barx1^−/−^* mouse embryos is abnormally elongated and the point of esophago-tracheal separation shows marked caudal displacement, resulting in a common foregut tube that is similar to human congenital tracheo-esophageal fistula and explains neonatal lethality. Moreover, the *Barx1^−/−^* esophagus displays molecular and cytologic features of respiratory endoderm, phenocopying abnormalities observed in mouse embryos with activated ß-catenin. The zone of canonical Wnt signaling is abnormally prolonged and expanded in the proximal *Barx1^−/−^* foregut. Thus, as in the developing stomach, but distinct from the spleen, Barx1 control of thoracic foregut specification and tracheo-esophageal septation is tightly associated with down-regulation of adjacent Wnt pathway activity.

## Introduction

The primitive gut develops from the union of definitive endoderm with the splanchnic mesoderm, first appearing as a fold on the ventral surface of unturned mouse embryos. By the end of embryonic day (E) 8, the anterior gut tube forms the foregut diverticulum, which originates from a small groove in the ventral midline endoderm and will differentiate into most of the oral cavity, pharynx, esophagus, and respiratory tract. Between E9 and E9.5, the anterior foregut narrows to form the prospective esophagus, coinciding with appearance on the ventral aspect of the oropharyngeal floor of the laryngo-tracheal groove, which extends to form the trachea [1], [2]. Reciprocal interactions between the endoderm and its adjoining mesenchyme direct patterning, morphogenesis, and maturation of these foregut-derived tissues [3], [4], [5]. Tracheo-esophageal fistula and esophageal atresia (EA/TEF), congenital defects that occur in 1 per 2,000 to 4,000 live human births, often in association with other digestive tract anomalies [6], reflect errors in patterning and morphogenesis of the anterior (thoracic) foregut tube.

Various secreted signals, including Sonic hedgehog (Shh), Fibroblast growth factor (FGF) 10 and FGF receptor 2, Bone morphogenetic protein (BMP)-4 and Noggin, and Wnts are implicated in foregut patterning because disruption of the corresponding genes leads to foregut development defects, including the EA/TEF complex [4]. In particular, Wnt signaling plays an important role in dorso-ventral patterning. Several Wnt ligands, including *Wnt2* and *Wnt2b*, are expressed, and *Axin2-lacZ* transgenic mice reveal Wnt activity, in the developing foregut between E9 and E10.5, when the esophagus and trachea are specified [7], [8]. Combined loss of *Wnt2* and *Wnt2b* or inactivation of ß-catenin (*Ctnnb1*) in the endoderm lead to failure in foregut separation, whereas activation of ß-catenin in the endoderm expands the respiratory domain in the dorsal foregut endoderm [7], [8].

The homeobox gene *Barx1* is highly expressed in the fetal mouse stomach mesenchyme, from where it directs differentiation of the overlying endoderm [3], [9]. In the prospective stomach, Barx1 is required for expression of secreted Wnt antagonists, such as secreted frizzled-related proteins (sFRPs) 1 and 2, which suppress local Wnt signaling to confer stomach-specific identity on the overlying endoderm [3]. Notably, the proximal *Barx1^−/−^* esophagus, which seemed initially to lie beyond the *Barx1* expression domain, showed radial asymmetry, with a typical squamous mucosa on one surface and a ciliated cuboidal epithelium on the other [10]. We reasoned that this cuboidal epithelium may represent mis-localized cells of the respiratory epithelium and that occult *Barx1* expression in the proximal foregut mesenchyme may influence esophageal differentiation, much as it does in the stomach. Furthermore, we noted that in the immature endoderm, an activating ß-catenin mutation, which simulates canonical Wnt signaling, expands respiratory epithelium at the expense of esophageal fate [7], [8]. Accordingly, if Barx1 were to suppress Wnt signaling not just in the stomach but also in the proximal foregut, the esophageal defects in *Barx1^−/−^* embryos might represent the consequence of excessive Wnt activity. We therefore hypothesized that Barx1 in the mesenchyme affects thoracic foregut development by suppressing regional endodermal Wnt activity.

To test this model, we examined *Barx1* expression in foregut development, characterized the morphogenic and molecular defects in *Barx1^−/−^* foregut, and assessed Wnt pathway activation. Our studies reveal a previously unappreciated role for mesenchymal Barx1 in patterning the entire foregut by suppressing Wnt/β-catenin signaling in the adjoining endoderm. Beyond providing a cohesive mechanism for Barx1 effects on gut endoderm development, our study reveals a molecular pathway that may contribute to proximal tracheo-esophageal septation defects in humans.

## Results

### Dynamic Barx1 expression in foregut mesenchyme

**Figure 1:**
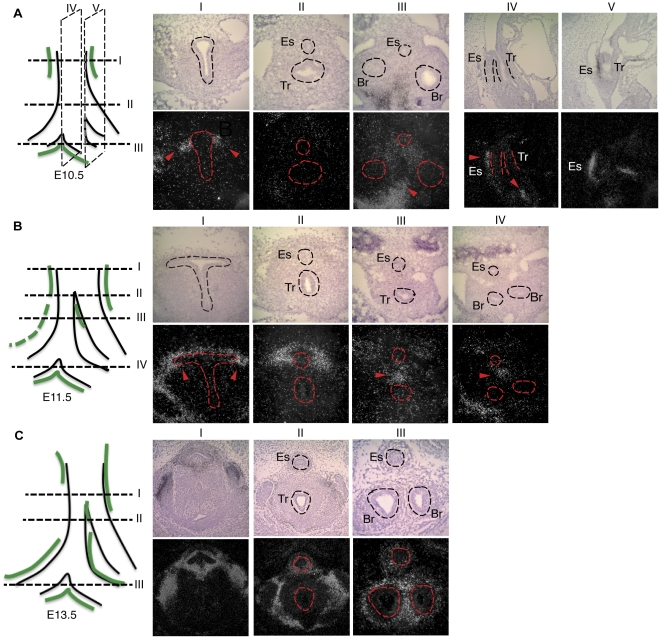
*Barx1* expression in proximal (thoracic) foregut development. Radioactive *Barx1* in situ hybridization at E10.5 (**A**), E11.5 (**B**) and E13.5 (**C**). Upper panels show bright-field and lower panels show dark-field images; in each case, dorsal is on top and ventral on the bottom. Images I to III in A and C and I to IV in B represent cross-sections at the axial levels indicated in the diagrams at the left, where the green shading summarizes sites of Barx1 expression; images A-IV and A-V show sagittal sections of E10.5 embryos, with medial to the left and lateral to the right. Sense probes hybridized at the same time gave no signal. Dashed lines mark developing structures Es, esophagus; Tr, trachea; Br, bronchi. Red arrowheads point to selected sites of abundant *Barx1* mRNA expression: dorsolateral mesenchyme around the laryngotracheal groove and upper esophagus (A-I, A-IV, A-V, B-I), mesenchyme separating the prospective esophagus and trachea (B-III, B-IV), and mesenchyme between the mainstem bronchi (C-III). Data were obtained on 2 embryos at each stage.

Because Barx1 expression is especially prominent in the stomach, which is severely malformed in *Barx1^−/−^* mice [3], we initially assumed that the esophageal defects were secondary [10], although they were not readily explained on that basis. To consider the possibility of direct effects on esophageal development, we re-examined Barx1 expression in the proximal digestive tract. At E10.5, *Barx1* mRNA is expressed symmetrically in mesenchyme flanking the dorsal foregut at the level of the laryngo-tracheal groove, limited to the level at which separation of the trachea and esophagus initiates. Caudal to this region, at the level of the esophagus and bronchi, we observed strong *Barx1* expression in the mesenchyme medial to both mainstem bronchi (Fig. 1A). Sagittal tissue sections confirmed mesenchymal *Barx1* mRNA expression surrounding the anterior dorsal foregut and medial to the bronchial stems (Fig. 1 A-IV and A-V).

*Barx1* in situ hybridization signals increased by E11.5 in the mesenchyme of the laryngotracheal groove and upper esophagus (Fig. 1B). Posteriorly, *Barx1* mRNA concentrated in the mesenchyme between the lower esophagus and trachea, although the signal extended caudally to the mesenchyme between the esophagus and mainstem bronchi (Fig. 1B). This expression pattern persisted at E13.5, with further increase in the signal intensity. At the level of the laryngeal aditus, *Barx1* mRNA was confined to mesenchyme surrounding the proximal esophagus and thyroid gland. The posterior expression domain shifted toward dorsal and medial mesenchyme; abundant signals were present around the mainstem bronchi (Fig. 1C), more prominent in the medial than in the lateral domains.

Thus, although *Barx1* levels in the developing thoracic foregut are lower than in the branchial arches or stomach, these data reveal expression in cells surrounding the esophagus, trachea, and mainstem bronchi. Expression is restricted to the mesenchymal compartment and excluded from the nascent aerodigestive epithelium.

### Barx1 deletion leads to defective foregut septation and to expansion and mislocalization of respiratory endoderm

**Figure 2:**
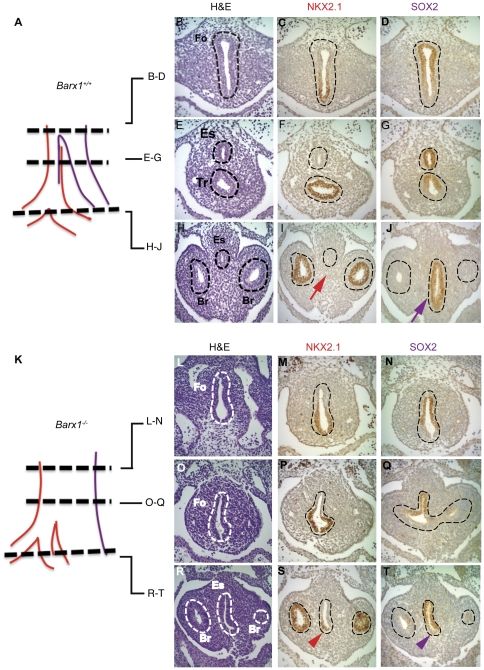
Anatomy and marker expression in E10.5 thoracic foregut of E10.5 *Barx1^+/+^* (A–J) and *Barx1^−/−^* (K–T) embryos. Anatomic structures and the domains of Nkx2.1 (red) and Sox2 (purple) expression are depicted in diagrams in A and K, where axial levels of each row of micrographs are marked with dotted lines. The left column shows hematoxylin and eosin-stained tissue sections, with dashed lines demarcating the undivided foregut (Fo), esophagus (Es), trachea (Tr) and mainstem bronchi (Br); these dashed lines carry over into the immunohistochemical micrographs for NKX2.1 (middle) and SOX2 (right column). In each image, dorsal is on top and ventral on the bottom. The results reveal ectopic NKX2.1 (red arrowhead in S) and loss of SOX2 (purple arrowhead in T) expression in the ventral endoderm of the undivided *Barx1^−/−^* foregut, corresponding to tissue that shows SOX2 (purple arrow in J) but no Nkx2.1 (red arrow in I) expression in *Barx1^+/+^* littermates. Results are representative of experiments with 3 embryos of each genotype.

To investigate the role of Barx1 in anterior foregut development, we assessed tissue morphology and expression of the transcription factors Nkx2.1, Sox2 and p63 in mutant and control embryos. The homeodomain protein Nkx2.1 is one of the earliest markers of developing respiratory endoderm, including trachea and lungs [11]; Sox2 is a useful marker of the dorsal foregut, esophagus, and anterior stomach [12], whereas p63 is required for development of many stratified epithelia, including that of the esophagus [12], [13]. Compared to wild-type controls, in which the esophagus and trachea were well separated by E10.5 (Fig. 2E–G), *Barx1^−/−^* embryos showed contiguity of the Nkx2.1-expressing tracheal epithelium and Sox2-expressing esophageal epithelium in a single luminal structure, indicating failure of foregut septation (Fig. 2 M–N, P–Q, S–T). This pattern of marker expression was well reflected in the tissue morphology, as *Barx1^−/−^* embryos showed a single elongated foregut tube instead of distinct tracheal and esophageal structures; absence of separation was evident over the full length of the thoracic foregut, extending caudal to the coronal level of the mainstem bronchi (Fig. 2 L, O, R). The presence of a strong Nkx2.1 signal in the bronchial epithelium points to intact lung bud formation and lung endoderm specification in *Barx1^−/−^* embryos. Furthermore, Nkx2.1-expressing lung progenitors, present in the ventral thoracic foregut, continued into the esophagus-stomach junction (Fig. 2S, red arrowhead). By contrast, Sox2-expressing esophageal endoderm was confined to the dorsal surface and excluded from the ventral esophagus and stomach (Fig. 2T, purple arrowhead). The stratified epithelial marker p63, present in wild-type E10.5 esophagus, was undetectable in Nkx2.1-expressing dorsal foregut cells in *Barx1^−/−^* mutants (blue arrowhead in Fig. S1E), suggesting inadequate squamous cell differentiation.

**Figure 3:**
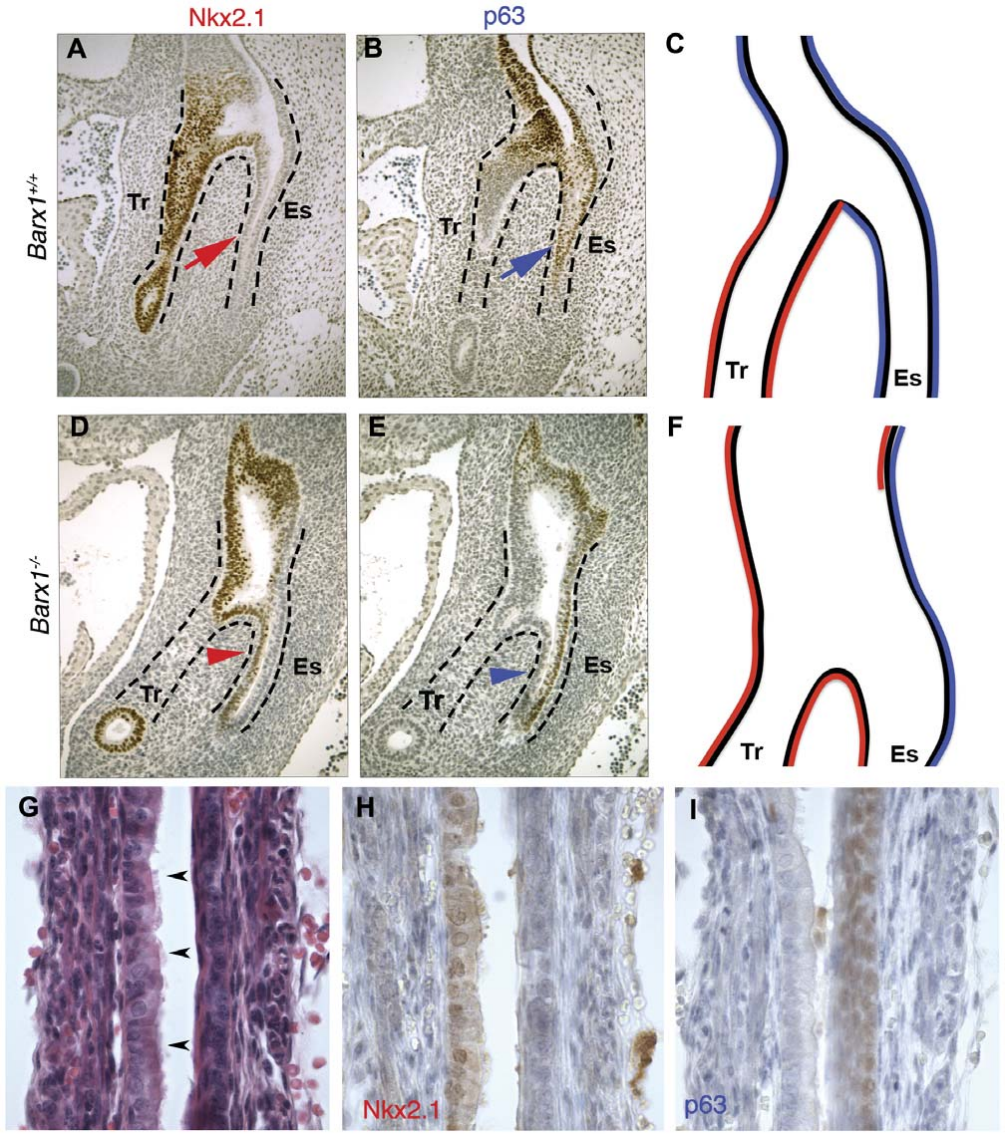
Molecular and histologic evidence for failure of tracheo-esophageal septation in the absence of Barx1. Expression of the respiratory marker NKX2.1 and the stratified epithelial marker p63 in the thoracic foregut of E13.5 *Barx1^+/+^* (A–C) and *Barx1^−/−^* (D–F) embryos. The sagittal tissue sections reveal an undivided rostral foregut, with NKX2.1-expressing respiratory epithelial cells (red arrowhead in D) replacing p63-expressing (blue arrow in B and blue arrowhead in E) squamous epithelial cells in the ventral esophageal endoderm. The results are interpreted in diagrams in C, F. (**G–I**) Histologic and immunologic demonstration of asymmetry in the lining of the distal esophagus in E19.5 *Barx1^−/−^* pups, with a stratified squamous epithelium on the dorsal surface and a columnar respiratory epithelium along the ventral surface (arrowheads in G). Immunostaining revealed mutually exclusive NKX2.1 expression in ventral cells (H) and p63 expression in the dorsal epithelium (I). The data represent results from 2 mutant embryos.

The persistence and sequelae of these defects were apparent in *Barx1^−/−^* embryos at E13.5. The common anterior foregut remained thick and elongated, reflecting persistent failure of septation (Fig. 3D–F). The ventral foregut showed high Nkx2.1 and absent p63 expression (arrowheads in Fig. 3D,E), whereas wild-type littermates expressed p63 but not Nkx2.1 in the corresponding region (arrows in Fig. 3A,B). Thus, Nkx2.1-expressing cells extended aberrantly into the ventral esophageal endoderm, a region depleted of p63-expressing cells (Fig. 3D,E). Taken together, these results reveal molecular features of a respiratory endoderm progenitor in the ventral endoderm of the mid-gestation *Barx1^−/−^* esophagus. As we reported previously but could not then explain [10], even in late gestation (E19) the ventral surface of the esophagus carries a ciliated, columnar epithelium, distinct from the stratified epithelium of the dorsal surface (arrowheads in Fig. 3G). Immunostaining revealed mutually exclusive NKX2.1 expression in ventral cells and p63 expression in the dorsal epithelium (Fig. 3H,I). These findings indicate that Barx1 is necessary for tracheo-esophageal septation and for proper localization of squamous esophageal and columnar respiratory epithelia originating in the undifferentiated foregut.

### inhibits canonical Wnt signaling in the foregut endoderm

During stomach development Barx1 acts on Wnt signaling non-cell autonomously, by regulating the expression of secreted Wnt antagonists [3]. Although this precedent provided a clue for Barx1 mechanisms in the proximal foregut, Barx1 also exerts a potent, Wnt-independent effect in spleen development [10], revealing diverse actions. However, the defects in *Barx1^−/−^* thoracic foregut closely resemble those reported in *Ctnnb1^(ex3)flox^;Shh^cre^* mutants, where constitutive ß-catenin activation simulates unchecked Wnt signaling in the developing foregut endoderm [7]. We therefore postulated that the defects in proximal foregut development in *Barx1^−/−^* embryos might also result from a failure to suppress regional Wnt pathway activity. Indeed, in introducing the Axin2^LacZ^ Wnt-reporter [14] gene on the *Barx1^−/−^* background, we had previously observed prominent and aberrant ß-galactosidase activity in the shortened esophagus of E17.5 embryos [10]. However, we did not appreciate the significance of this finding for esophageal development at that time because high Barx1 expression in the prospective stomach had masked the esophageal expression we now report in Figure 1. To corroborate this result and to localize aberrant Wnt pathway activity in detail, we crossed *Barx1^+/−^* mice with *TOPGAL* transgenic mice, which also express *LacZ* in response to Wnt stimulation and hence localize canonical Wnt signaling faithfully in embryos [15]. Any role for Barx1 in suppressing nearby Wnt signaling should reflect as increased LacZ reporter activity in *Barx1^−/−^;TOPGAL* endoderm adjacent to the sites of mesenchymal Barx1 expression.

**Figure 4:**
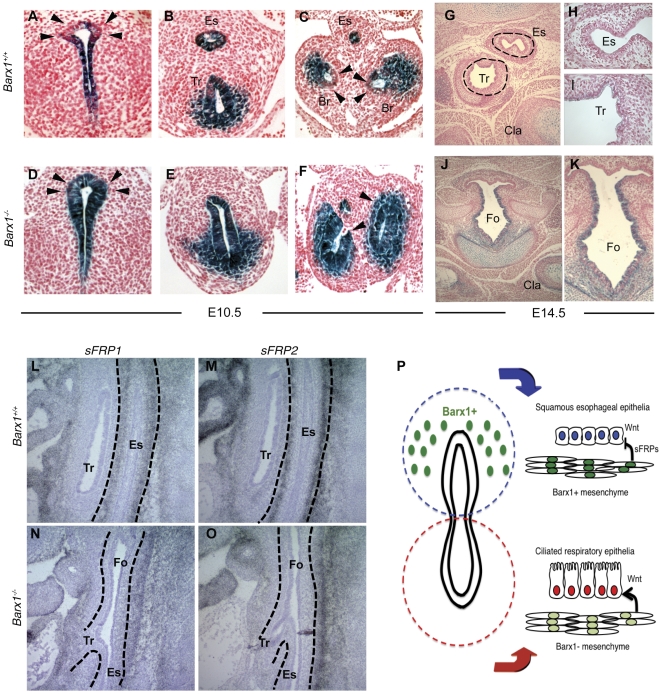
Aberrant activity of the canonical Wnt pathway in *Barx1^−/−^* thoracic foregut derivatives, resulting from reduced expression of secreted Wnt antagonists. (**A–K**) ß-galactosidase staining (blue) in tissues from *Barx1^+/+^; TOPGAL* (A–C, G–I) and *Barx1^+/+^;TOPGAL* embryos (D–F, J–K) at E10.5 (A–F) and E14.5 (G–K). Wnt activity persists in the *Barx1^−/−^* dorsal foregut (D), medial bronchial endoderm (F), and adjoining mesenchyme (E) at E10.5, as well as the undivided foregut at E14.5 (J,K), areas with clearly diminished ß-galactosidase/Wnt activity in *Barx1^+/+^* littermates. The domains of aberrant Wnt activity in mutant embryos correspond to sites adjacent to mesenchymal Barx1 expression shown in Figure 1. Images in H, I and K show higher magnification of tubular structures shown in G and J. (**L–O**) In situ hybridization analysis of secreted Wnt antagonists sFRP1 (L, N) and sFRP2 (M, O) in the esophageal mesenchyme of E13.5 wild-type (L, M) and *Barx1^−/−^* (N, O) embryos; dotted lines demarcate the esophagus. Caudal displacement of the tracheo-esophageal bifurcation is again evident in these sagittal images and persistence of signal outside the esophagus highlights the anatomic restriction of reduced sFRP expression. The data represent results from 3 (ß-galactosidase staining) or 2 (sFRP expression) embryos of each genotype. Es, esophagus; Tr, trachea; Br, bronchi; Cla, clavicle; Fo, foregut. (**P**) Model for the role of Barx1 and Wnt signaling in differentiation of thoracic foregut structures and epithelia. Absence of Barx1 and ensuing excessive Wnt signaling result in differentiation of NKX2.1+ respiratory epithelium at the expense of Sox2+ p63+ squamous esophageal epithelium.

*Barx1^−/−^;TOPGAL* embryos showed robust ß-galactosidase activity in E10.5 anterior dorsal foregut endoderm (Fig. 4D, arrowheads), a region that showed little to no signal in *Barx1^+/+^; TOPGAL* embryos (Fig. 4A, arrowheads). Additionally, whereas Wnt/ß-galactosidase activity in control mainstem bronchi was confined to the lateral endoderm and mesoderm but excluded from the medial tissue, *Barx1^−/−^;TOPGAL* embryos showed intense circumferential reporter gene activity (arrowheads in Fig. 4C,F); even the adjoining mesenchyme showed heightened Wnt pathway activity (Fig. 4B,E). These areas correspond precisely to those regions where Barx1 is selectively expressed in the adjacent mesenchyme (compare, for example, Fig. 4A with in situ hybridization results in Fig. 1 A–I, B–I, and B-II).

Wnt signaling in the developing proximal foregut is transient, and completely attenuated by E14.5 in wild-type mice (Fig. 4G–I). By contrast, at E14.5 the unseparated thoracic foregut in *Barx1^−/−^; TOPGAL* embryos showed persistent Wnt pathway activity (Fig. 4J,K), reflecting a consequence of Barx1 loss. ß-galactosidase activity was comparable in control and *Barx1^−/−^;TOPGAL* bone and cartilage, including clavicles, vertebral bodies and larynx (Fig. 4G,J), tissues that lack Barx1 expression. These data suggest that Barx1 normally suppresses Wnt signaling in adjoining foregut tissues. Considered in the light of known Wnt pathway roles in these tissues [7], [8], [10], our results implicate a Wnt-dependent mode of action for Barx1 in foregut development (Fig. 4B), similar to its action in the embryonic stomach and distinct from that in the developing spleen.

Barx1 effects on stomach development are mediated, at least in part, through secreted Frizzled-related proteins (sFRPs) that antagonize Wnt pathway activity [3]. To determine if this is also the case in the esophagus, we used RNA in situ hybridization to examine sFRP expression in *Barx1^−/−^* embryos. Compared to wild-type littermates, which express both transcripts robustly in the esophageal mesenchyme (Fig. 4L,M), mutant E13.5 embryos showed virtual absence of *sFRP1* and *sFRP2* mRNAs (Fig. 4N,O). This loss seems restricted to the esophagus, as expression in adjacent neural structures is unaffected. Thus, Barx1 inhibition of nearby Wnt signaling may normally occur through regulated expression of sFRPs, in whose absence Wnt signaling remains aberrantly active (Fig. 4P).

## Discussion

Foregut development and the alternative specification of ventral respiratory and dorsal digestive endoderm depend on the coordinated actions of several signaling pathways. For example, *Shh*-null and *Gli2/Gli3*-mutant mouse embryos show severe foregut defects, including failure of tracheo-esophageal septation and delayed formation of lung buds [16], [17]. Loss of Fgf10 or its receptor Fgfr2 also results in absence of lung buds but tracheal specification is intact [18], [19]. Wnts 2 and 2b are expressed in the ventral foregut mesenchyme, where *Axin2-lacZ* activity reveals Wnt pathway activity. Activation of ß-catenin in the developing foregut endoderm expands the domain of *Nkx2.1* expression into dorsal foregut endoderm, whereas depletion of Wnts 2 and 2b or conditional inactivation of ß-catenin abrogate *Nkx2.1* expression, associated with tracheal and lung agenesis [7], [8]. We report here that mouse embryos lacking the mesenchymal homeodomain transcription factor Barx1 fail to separate the esophagus and trachea properly, leaving a single foregut tube that is dominated ventrally by a Nkx2.1+ ciliated columnar respiratory epithelium. Our findings fit well within the emerging schema of pathways in anterior foregut development and implicate Barx1 as a regulator of Wnt signaling, responsible in part for limiting the duration and spatial extent of Wnt pathway activity in the foregut endoderm and adjacent mesenchyme (Fig. 4P).

Barx1 suppression of Wnt signaling, most likely through expression of the secreted Wnt antagonists Sfrp1 and Sfrp2, is known to specify stomach endoderm [3]. Barx1 is abundantly expressed in early mouse stomach mesenchyme and branchial arches, but we also identified expression in dorsal foregut mesenchyme, in cells located between the prospective esophagus and trachea, and in cells surrounding the mainstem bronchi, especially along the medial aspect. This expression coincides precisely with areas of progressive decline in Wnt activity during development of the adjacent endoderm, supporting the idea that Barx1 might repress Wnt signaling throughout the foregut. Thus, although Wnt signaling is required early in foregut development, its attenuation may be equally necessary to ensure proper segregation of tissues and Barx1 helps achieve this attenuation. At a minimum, the prominent foregut defects that occur in the absence of Barx1 are associated with residual or increased Wnt pathway activity. This persistent activity may suffice for the shift toward respiratory epithelium, similar to observations in mouse embryos with active ß-catenin in this tissue [7], [8]. The sum of data hence suggests that Barx1 normally helps establish esophageal fate at the expense of respiratory epithelium through suppression of Wnt activity in the adjacent ventral endoderm. This conclusion is consistent with the idea that mesenchymal signals constitute the principal force to direct differentiation of unspecified endoderm.

BMP4 is expressed in the ventral foregut mesoderm and probably signals to both the mesoderm and the foregut endoderm, whereas the dorsal foregut endoderm, notochord and floor plate express the BMP antagonist Noggin. Analysis of *Nog*-null mice suggests that BMP signaling regulates initial dorsal-ventral patterning of the foregut and its separation into the dorsal esophagus and ventral trachea [2], [20]. Although BMP signaling plays additional roles in esophagus and forestomach development [21], the downstream mechanisms are unknown. Borrowing from findings in other tissues, our data raise the possibility that BMP and Noggin regulate foregut patterning in part through Barx1. In jaw primordia and dental mesenchyme, where *Barx1* is abundantly expressed, BMP signaling reduces *Barx1* expression [22], [23]. Furthermore, the TEF that develops in *Nog*-null mutant mice is believed to reflect unopposed BMP4 activity in the ventral foregut and, similar to *Barx1^−/−^* mutants, shows persistent *Nkx2.1* expression in a common foregut tube [2]. If BMPs also down-regulate Barx1 in foregut mesenchyme, then increased BMP activity in *Nog*-null mutants may culminate in TEF by suppressing dorsal foregut Barx1 levels. Thus, we speculate that *Nog*-null mutant mice have altered *Barx1* expression and aberrant Wnt activity in the thoracic foregut.

In summary, this study reveals an unexpected role for Barx1 in development of the proximal (thoracic) foregut. We identified severe tracheo-esophageal defects in *Barx1^−/−^* embryos and provide a satisfying explanation that mirrors the role of Barx1 as a negative regulator of Wnt signaling in stomach morphogenesis and specification. The results indicate that acquisition or maintenance of a squamous epithelium in the ventral esophagus depends on Barx1 function and related attenuation of Wnt pathway activity.

## Materials and Methods

### Experimental animals

Male *Barx1* ^+/−^ mice on a 129/Sv genetic background were back-crossed with heterozygote C57BL/6 females and we studied most *Barx1* mutant pups after at least five back-crosses. *TOPGAL* transgenic mice and strain-matched CD1 controls were purchased from Jackson Laboratories (Bar Harbor, ME); *Barx1^−/−^*;*TOPGAL* mice were generated by interbreeding. Animals were handled according to protocol number 03-132, approved by the Dana-Farber Cancer Institute's Animal Care and Use Committee. The morning following vaginal plugging was regarded as day 0.5 of gestation.

### Histology and immunohistochemistry

After overnight fixation in Bouin's solution or 4% paraformaldehyde, whole embryos or isolated organs were dehydrated, embedded in paraffin, and sections of 5–6 µm thickness were prepared. Hematoxylin and eosin (H&E) staining followed routine methods. For antigen retrieval prior to immunostaining, specimens were heated to125°C in 10 mM Na citrate buffer (pH 6.0) in a decloaking chamber (Biocare Medical, Concord, CA), followed by cooling for 60 min at room temperature. Endogenous peroxidases were inhibited by treatment in methanol containing 0.5% H_2_O_2_ for 30 min. After blocking with normal goat serum, samples were incubated for 16 h at 4°C with NKX2.1 (1∶100, Thermo Scientific), p63 (1∶100, Thermo Scientific) or SOX2 (1∶1000, Chemicon) antibodies (Ab), washed, incubated with biotinylated goat anti-mouse, anti-rabbit or anti-rat IgG, and treated with avidin-biotin-peroxidase complex (Vector Laboratories, Burlingame, CA). Color reactions were developed with diaminobenzidine hydrochloride solution (Sigma-Aldrich).

### β-galactosidase staining

Pregnant dams were sacrificed and embryos treated with a LacZ (β-galactosidase) staining protocol that gave no background in non-transgenic animals. Mouse embryos were isolated in Ca^2+^- and Mg^2+^-free Hanks' Balanced Salt Solution (Invitrogen, Carlsbad, CA), fixed for 15 min with 4% paraformaldehyde in phosphate-buffered saline (PBS), washed 3 times in PBS, and incubated for 9-10 hours at 37°C in staining solution containing PBS (pH 7.2), 1 mg/ml 5-bromo-4-chloro-3-indoyl-β-D-galactoside, 5 mM K_3_Fe(CN)_6_, 5 mM K_4_Fe(CN)_6_·3H_2_O, 1 mM MgCl_2_, 0.01% sodium deoxycholate, and 0.02% NP40. Tissues were then dehydrated, embedded in paraffin, cut in 8-µm sections, and counterstained with nuclear fast red.

### RNA in situ hybridization

8-µm thick paraffin sections of paraformaldehyde-fixed CD1 or Barx1 mutant mouse embryos were hybridized with ^35^S-UTP-radiolabeled Barx1, sFRP1 and sFRP2 antisense riboprobes as described [3]. Slides were counterstained with hematoxylin (Fluka) and examined by dark-field microscopy. Higher levels of sFRP1 and sFRP2 mRNAs are shown with bright-field microscopy to aid in transcript localization.

## Supporting Information

Figure S1 **Differing domains of expression of the stratified epithelial (esophageal) marker p63 in E10.5** ***Barx1^+/+^*** **(A–C) and** ***Barx1^−/−^*** **(D–F) thoracic foregut derivatives.** Axial levels, rostral to caudal, are depicted by dashed lines in the corresponding diagrams. Dotted lines within the micrographs demarcate the undivided foregut (Fo), esophagus (Es), trachea (Tr), and mainstem bronchi (Br). In each image, dorsal is on top and ventral on the bottom. The results reveal stronger p63 staining in *Barx1^+/+^* squamous esopheal epithelium (B) than in the undivided *Barx1^−/−^* foregut (blue arrowhead in E).
(TIF)
