# Supplementary figures and images for: Barx1-Mediated Inhibition of Wnt Signaling in the Mouse Thoracic Foregut Controls Tracheo-Esophageal Septation and Epithelial Differentiation
Source: PLoS One. 2011 Jul 22;6(7):e22493. doi: 10.1371/journal.pone.0022493 (PMC3142160; doi:10.1371/journal.pone.0022493)

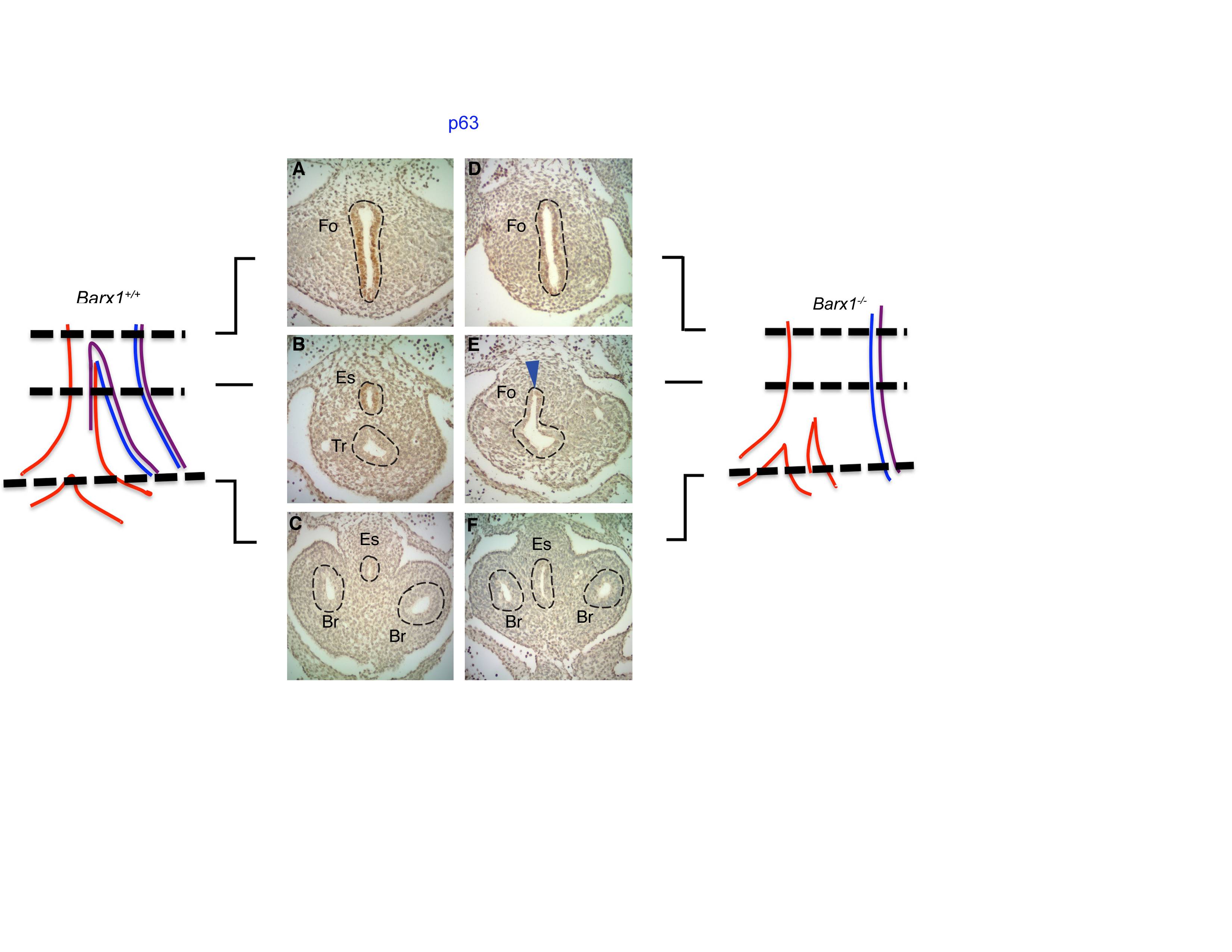

Supplement: Figure S1 — Differing domains of expression of the stratified epithelial (esophageal) marker p63 in E10.5 Barx1+/+ (A–C) and Barx1−/− (D–F) thoracic foregut derivatives. Axial levels, rostral to caudal, are depicted by dashed lines in the corresponding diagrams. Dotted lines within the micrographs demarcate the undivided foregut (Fo), esophagus (Es), trachea (Tr), and mainstem bronchi (Br). In each image, dorsal is on top and ventral on the bottom. The results reveal stronger p63 staining in Barx1+/+ squamous esopheal epithelium (B) than in the undivided Barx1−/− foregut (blue arrowhead in E). (TIF) [file pone.0022493.s001.tif]
